# Neuroimaging in posterior cortical atrophy: an integrative review for clinicians and radiologists

**DOI:** 10.3389/fneur.2025.1631245

**Published:** 2025-08-06

**Authors:** Fábio Henrique De Gobbi Porto, Mari Nilva Maia da Silva, Célia Sarah Gava Jorge Bravo, Gislaine Cristina Lopes Machado Porto, Wesley Américo Bergamin Granado de Paula, Sérgio José Silva Fernandes, Gilberto Miyazaki Otta, Gordon Plant

**Affiliations:** ^1^Laboratory of Psychiatric Neuroimaging (LIM-21) and Old Age Research Group (PROTER), Department and Institute of Psychiatry, Faculty of Medicine, University of São Paulo, São Paulo, Brazil; ^2^University College London Queen Square Institute of Neurology, London, United Kingdom; ^3^Division of Neuropsychiatry, Hospital Nina Rodrigues, São Luís, Brazil; ^4^University Hospital of Londrina, State University of Londrina (UEL), Londrina, Brazil; ^5^Division of Neuroradiology, Alphasonic Group, Londrina, Paraná, Brazil; ^6^Division of Head and Neck Radiology, Alphasonic Group, Londrina, Paraná, Brazil; ^7^Department of Diagnostic Imaging, A. C. Camargo Cancer Center, São Paulo, Brazil; ^8^Division of Neurology, Hospital São Domingos, São Luís, Brazil

**Keywords:** posterior cortical atrophy, magnetic resonance imaging, FDG (18F-fluorodeoxyglucose)-PET/CT, tau PET, Alzheimer disease, visual cortex

## Abstract

Posterior cortical atrophy (PCA) is most frequently an atypical variant of Alzheimer’s disease that primarily manifests through visual symptoms of cortical origin. This review provides a comprehensive overview of neuroimaging in PCA, addressing key anatomical and clinical aspects as well as characteristic findings across different imaging modalities. It is intended for non-radiologist clinicians and radiologists who may encounter cognitive dysfunction less frequently, aiming to enhance early recognition and accurate interpretation of imaging studies.

## Highlights

- *Posterior cortical atrophy* is most often a visual variant of Alzheimer’s disease, with predominant involvement of occipitoparietal and occipitotemporal cortices.
- *Neuroimaging evaluation*, including MRI and PET, is fundamental for early detection and diagnosis.
- *Dorsal and ventral visual streams* explain the clinical heterogeneity in PCA presentations.
- *Visual rating scales* (such as the Koedam scale) and *automated volumetry* assist in quantifying regional atrophy.
- *FDG and Tau PET imaging* provide the closest correlation with clinical symptoms. Amyloid PET does not reflect clinical localization.

## 1 Introduction

The first description of a focal, visuospatial predominant form of Alzheimer’s disease (AD) was made by Cogan (1). Three years later, Benson et al. (2) proposed posterior cortical atrophy (PCA) as a clinico-radiological syndrome characterized by relatively isolated, progressive dysfunction of posterior cortical regions (2). Clinically, PCA manifests with features of Bálint’s syndrome (simultanagnosia, optic ataxia, ocular apraxia), Gerstmann’s syndrome (acalculia, agraphia, right–left disorientation, finger agnosia), as well as alexia, visual agnosia, and transcortical sensory aphasia, accompanied by imaging evidence of occipitoparietal and occipitotemporal atrophy (3).

Although episodic memory, executive function, and language abilities are relatively preserved in the early stages, they progressively deteriorate as the disease advances, converging toward the clinical profile of typical Alzheimer’s dementia (2, 4). AD is indeed the most common underlying pathology in PCA, accounting for 62 to 100% of autopsy-confirmed cases (5–8), with an atypical distribution of lesions: PCA patients exhibit a higher burden of amyloid plaques and, more notably, neurofibrillary tangles in posterior cortical regions (9), and most particularly in parietal cortex (10).

The strong pathological association between PCA and AD — even stronger than that seen in the amnestic presentations — has led PCA to be recognized as a canonical atypical (visual) variant of AD (11). PCA accounts for approximately 5% of AD cases in memory clinics (12, 13), although diagnosis is often delayed and misdiagnosis is not rare (14).

Over the past two decades, the phenotype of PCA has been better characterized. Visual field defects, which were thought to be rare, are now recognized as relatively common (8, 15, 16). Furthermore, a consensus classification has been proposed to distinguish between “pure PCA” and “PCA-plus” phenotypes, aiding in the estimation of the likelihood of non-AD pathology (15).

Given the advent of disease-modifying therapies for AD — although at the time of writing not explicitly approved for PCA — accurate and early diagnosis of this syndrome is crucial (17). The diagnosis of PCA involves a two-step process: clinical characterization followed by neuroimaging assessment. Neuroimaging remains challenging in clinical practice, as general neurologists may not always recognize subtle changes in visual regions of the brain, and general radiologists may be unfamiliar with complex visual syndromes.

This paper introduces a fresh perspective through the integration of functional neuroanatomy with radiological interpretation and structured visual neuroimaging analysis for practical use. The article establishes detailed connections between dorsal and ventral visual stream dysfunction and particular neuroimaging results while highlighting essential anatomical markers which standard clinical practice tends to miss. The authors support the use of validated grading scales (Koedam and GCA scales) together with volumetric methods for structured visual inspection to improve both early and accurate diagnosis. The review targets general neurologists and radiologists by providing practical diagnostic guidance for atypical AD cases, which becomes crucial during the time of emerging disease-modifying therapies.

## 2 Anatomy and functional organization of the visual cortex

The human visual system can be defined as the apparatus that enables the psychological experience of visual perception. Visual perception results from a complex sequence of processes, including the reception, processing, and interpretation of visual information. The visual cortex is organized hierarchically, allowing basic visual features to be processed in early visual areas, progressing toward complex object, face, and visuo-spatial discrimination in higher-order cortical regions (18–20).

The primary visual cortex (V1) surrounds the calcarine sulcus on the medial surface of the occipital lobe. It is the first cortical area to receive visual input from the geniculocalcarine tract, which originates in the lateral geniculate nucleus. V1 is organized in a retinotopic manner and has a complex structure made up of vertical “hypercolumns” each of which receives input from a specific region of the retina of, predominantly, one eye. The neurons making up these columns are specialized in analyzing the orientation of luminance contrast defined borders, and also color and motion. From V1, information is transmitted along subcortical white matter to secondary visual areas (V2, V3, V4 and V5), which integrate visual signals and process the perception of motion, depth, color and contours (18, 19, 21). Damage to early visual areas, such as V1, results in cortical blindness although residual vision may be present as a result of parallel projections to subcortical and cortical regions. Of particular interest to PCA are the direct projections to the motion area (V5) which are likely to be the basis of the Riddoch phenomenon (preserved detection of motion) commonly seen in PCA (22)^1^.

Two major pathways emerge from the occipital lobe: the dorsal and ventral streams.

### 2.1 Dorsal stream–“where” pathway

The dorsal stream projects superiorly from the occipital cortex toward the posterior parietal lobe, particularly the parieto-occipital junction and intraparietal sulcus. This pathway is specialized in processing spatial location, movement direction, visuospatial attention, and visuomotor coordination, including eye movements (18, 19), whereas damage to more posterior parietal regions can lead to Bálint syndrome (23).

In PCA, dorsal stream impairment is associated with symptoms such as spatial disorientation, simultanagnosia, navigation difficulties, and optic ataxia (24).

### 2.2 Ventral stream–“what” pathway

The ventral stream also originates in occipital areas but extends anteriorly toward the inferior temporal lobe, involving structures including the fusiform gyrus (lateral occipitotemporal gyrus). This pathway is responsible for the recognition of shapes, colors, faces, and objects and plays a central role in visual recognition (18, 19).

In PCA, ventral stream degeneration may leads to clinical manifestations such as prosopagnosia, alexia, and visual agnosia (25). Recognizing the clinical-anatomical correlation between symptoms and ventral pathway degeneration is essential to differentiate PCA from other neurodegenerative syndromes.

## 3 Neuroimaging of the visuospatial system

### 3.1 Occipital lobe anatomy: complexity and clinical implications

The occipital lobe is the smallest and most posterior lobe of the cerebral hemispheres (excluding the insular lobe) (26, 27). Situated posterior to the temporal and parietal lobes, anterior to the occipital bone, and superior to the cerebellar tentorium, its most notable functional components are the primary and secondary visual cortices. Its anatomical boundaries include the parieto-occipital sulcus medially, and an imaginary vertical line extending from the pre-occipital notch to the parieto-occipital sulcus laterally (28). On the medial surface, it is bordered by the longitudinal fissure separating the two cerebral hemispheres (29). The lateral surface of the occipital lobe shows significant variability, marked by a complex sulcal and gyral pattern, complicating systematic identification in clinical practice (28, 30, 31). In contrast, major sulci such as the calcarine sulcus, parieto-occipital sulcus, occipitotemporal sulcus, and collateral sulcus are more consistently identifiable.

Koutsarnakis et al. (31) studied the variability of these structures in human brains, identifying the lateral occipital sulcus and intra-occipital sulcus in 100% of specimens (31). The transverse occipital sulcus was present in 88% of cases, while the inferior occipital sulcus appeared in only 15%. Based on these findings, the authors proposed a standardized anatomical nomenclature for clinical and educational use. These results were corroborated by Alves et al. (30), who emphasized the importance of the transverse occipital sulcus and intra-occipital sulcus as landmarks in the analysis of the lateral occipital convexity (30).

### 3.2 Key neuroimaging features in visuospatial system evaluation

**Figure 1:**
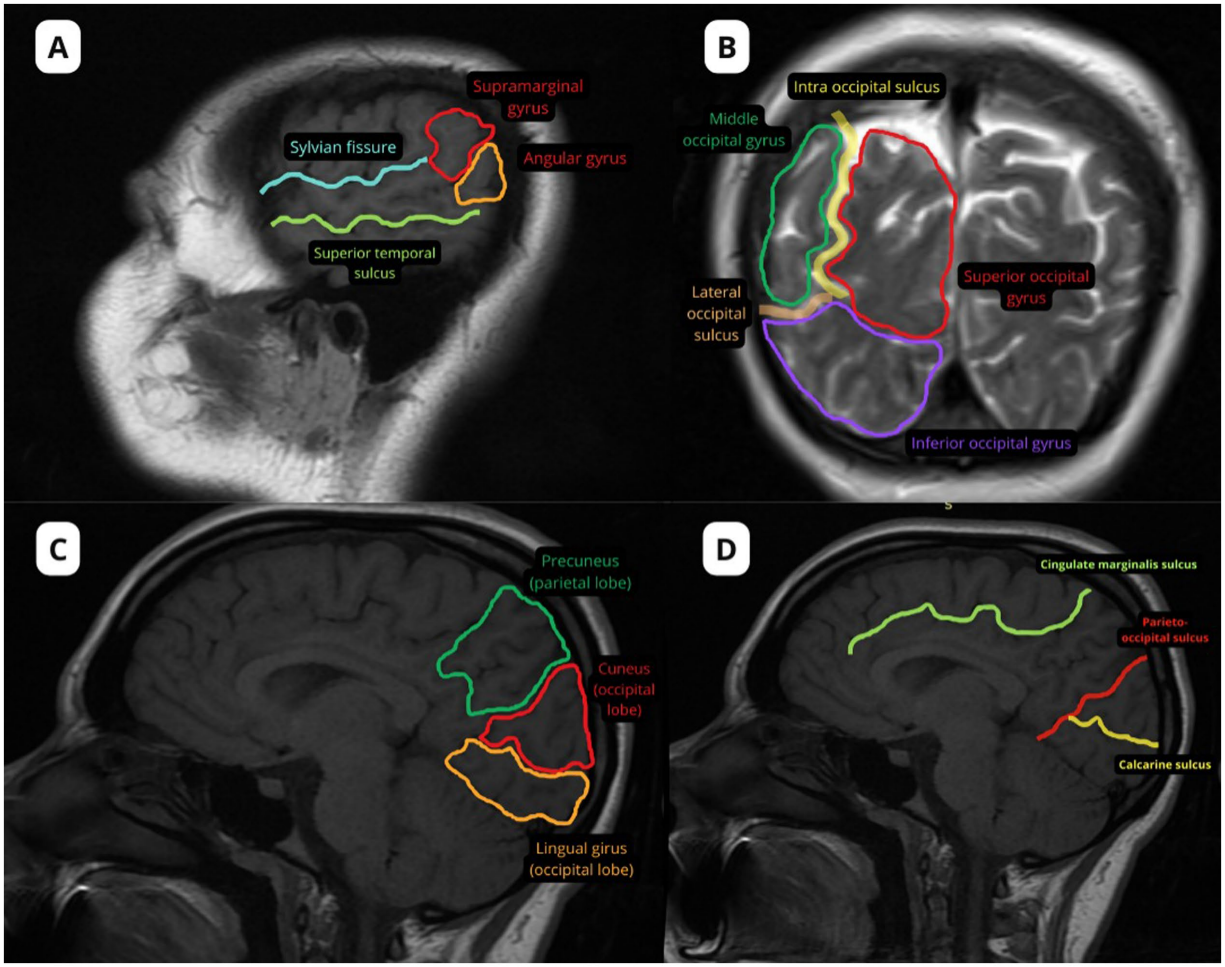
Main anatomical landmarks in the posterior region. Images were acquired using a GE scanner with a magnetic field strength of 1.5 Tesla. A pre-contrast T1-weighted sequence was used to highlight the alterations in **A,C,D**, while a T2-weighted sequence was used to highlight the alteration in **B**. **(A)** Supramarginal gyrus and angular gyrus; **(B)** Intra-occipital sulcus separating the superior occipital gyrus from the middle occipital gyrus, and lateral occipital sulcus separating the middle occipital gyrus from the inferior occipital gyrus; Precuneus (parietal lobe); cuneus and lingual gyrus (occipital lobe); **(D)** Marginal sulcus of the cingulate anterior to the precuneus, parieto-occipital sulcus separating the parietal lobe from the occipital lobe, and calcarine sulcus separating the cuneus from the lingual gyrus.

Magnetic resonance imaging (MRI) plays a critical role in detecting regional atrophy patterns suggestive of visuospatial dysfunction syndromes, such as PCA, most frequently caused by AD (3, 18, 19). Identifying anatomical landmarks on neuroimaging is essential for correlating lesion topography with clinical symptoms (Figure 1).

The primary visual cortex (V1), corresponding to Brodmann area 17, lies along the calcarine sulcus within the lingual and cuneus gyri, forming a well-defined band of gray matter (19, 32). The “calcarine” name refers to the “stria of Gennari,” a distinct histological band of white matter fibers visible to the naked eye (33). This is cortical layer IV, which identifies the major white matter input to cortex. The input comprises such a large number of fibers in V1 that, uniquely, the layer is visible with the naked eye and on imaging (34).

Secondary visual areas (V2 and V3), corresponding to Brodmann areas 18 and 19, surround V1 within the occipital lobe and are involved in higher-order visual processing, although they lack a clear anatomical boundary identifiable on MRI (28, 32).

## 4 Patterns of atrophy in posterior cortical atrophy

PCA can exhibit diverse clinical-anatomical patterns, including dorsal (occipitoparietal), ventral (occipitotemporal), and polar (predominantly occipital) presentations (3, 35). Early identification of atrophy and asymmetry (36) in these regions is critical for diagnosis.

On MRI, key anatomical landmarks for analysis include:

- Medial surface: parieto-occipital sulcus, calcarine sulcus; cuneus and lingual gyri.
- Inferior surface: lateral occipitotemporal sulcus, collateral sulcus; fusiform and parahippocampal gyri.
- Lateral surface: intra-occipital sulcus, lateral occipital sulcus, transverse occipital sulcus; superior, middle, and inferior occipital gyri.

For regions without established specific atrophy scales, it is recommended to refer to the Global Cortical Atrophy (GCA) scale (37, 38). We recommend using the GCA scale landmarks to systematically assess the main sulci and gyri of the occipital lobe, aiming to detect subtle atrophy and asymmetries. Applying a validated scale enhances the early detection of structural changes.

### 4.1 Definitions of key occipital sulci

Understanding the anatomy of occipital sulci is crucial for accurate structural MRI analysis in PCA:

- Intra-occipital sulcus: Continuation of the intraparietal sulcus beyond the parieto-occipital sulcus, easily identified on the brain convexity. It separates the superior and middle occipital gyri (30, 31).
- Lateral occipital sulcus (Middle Occipital Sulcus): A transverse sulcus located posterior to the parieto-occipital sulcus, emerging near the occipitotemporal junction on the superolateral cerebral surface (28, 30, 31, 39).
- Transverse occipital sulcus: Crosses the lateral occipital surface and assists in separating the superior occipital gyrus from the middle occipital gyrus (31).

## 5 Neuroimaging assessment using visual scales and volumetry

Advanced imaging techniques, including subjective visual rating and automated volumetry, have been increasingly utilized to localize and quantify atrophy patterns in PCA.

**Figure 2:**
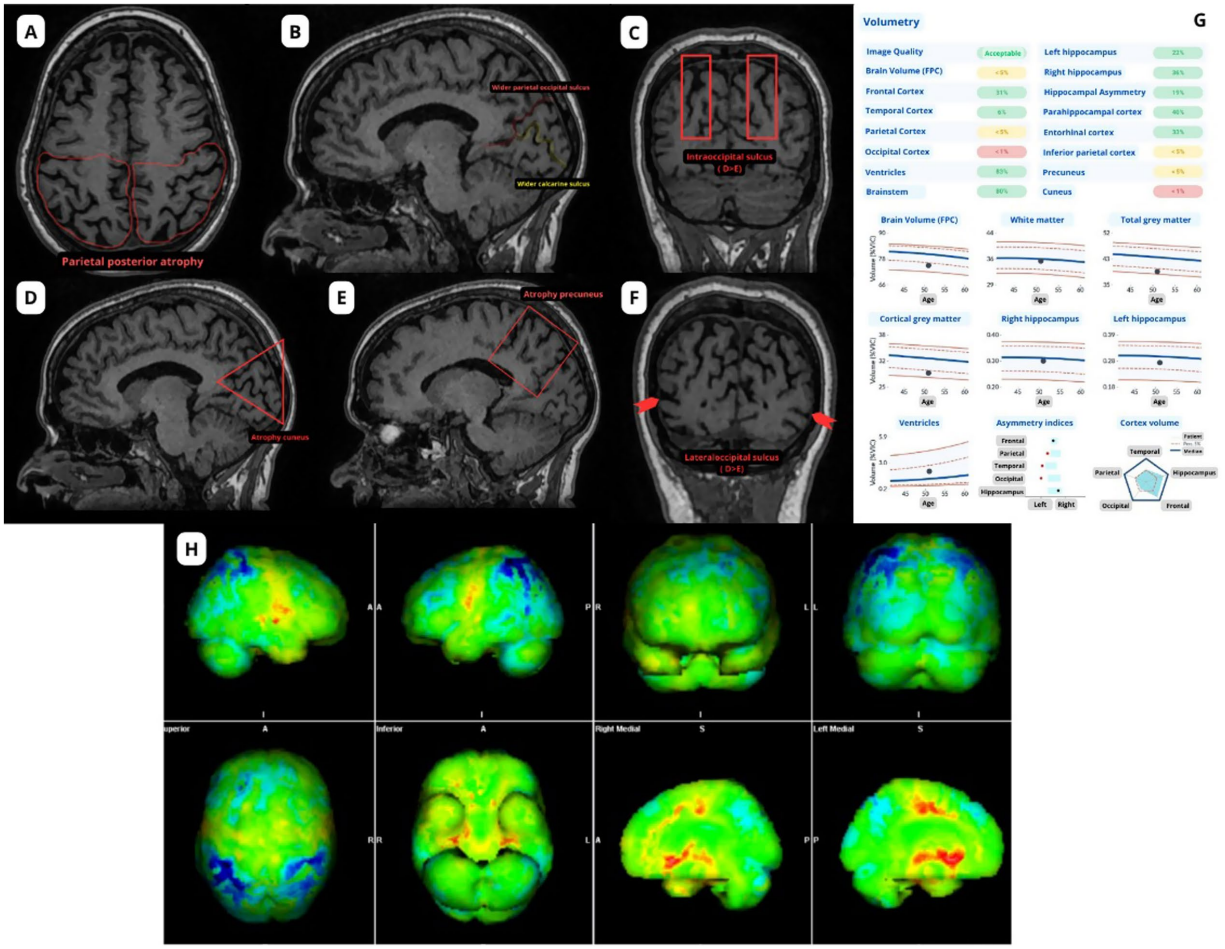
Main anatomical changes in posterior cortical atrophy. Images were acquired using a GE scanner with a magnetic field strength of 1.5 Tesla. A pre-contrast volumetric T1-weighted sequence was used to highlight the alterations from **(A)** to **(F)**, as well as for volumetric analysis. **(A)** Atrophy of the posterior parietal region; **(B)** Enlarged parieto-occipital sulcus and calcarine sulcus; **(C)** Right intra-occipital sulcus wider than the left; **(D)** Cuneus atrophy; **(E)** Precuneus atrophy; **(F)** Right lateral occipital sulcus wider than the left; **(G)** Volumetry; **(H)** FDG PET: Bilateral posterior parietal hypometabolism in a case of mild cognitive impairment due to synucleinopathy, with visuospatial deficits.

On visual inspection, marked volume reductions in the occipital and parietal lobes are typically observed, sometimes extending to specific temporal regions. Studies demonstrate predominant atrophy in parietal, parieto-occipital, and temporo-occipital areas, often asymmetrically favoring the right hemisphere, and involving the posterior cingulate gyrus, precuneus, and inferior parietal lobule (23, 35) (Figures 2A–F).

### 5.1 Visual rating scales

Visual rating scales offer an efficient and cost-effective approach to support clinical diagnosis (37). The *Koedam Posterior Atrophy Scale* evaluates atrophy across sagittal (posterior cingulate sulcus, parieto-occipital sulcus, precuneus), coronal (posterior cingulate sulcus, parietal lobe), and axial planes (posterior cingulate sulcus, parietal gyri), with scores ranging from 0 (no atrophy) to 3 (severe atrophy) (40). A modified visual scale emphasizing parieto-occipital sulcus widening has shown good reproducibility in differentiating PCA from typical AD (41). Careful evaluation of the parieto-occipital sulcus width is crucial for the early detection of occipital atrophy on MRI.

In addition to the Koedam posterior atrophy scale, the GCA scale can be used to assess diffuse or regional cortical atrophy in a systematic manner. The GCA is a semi-quantitative visual rating tool applied to axial T1-weighted MRI images, scoring cortical atrophy in frontal, temporal, parietal, and occipital lobes. Each region is graded from 0 (no atrophy) to 3 (severe atrophy), allowing a global assessment that is especially useful when specific rating scales for posterior regions are not available. This approach improves diagnostic sensitivity and reproducibility in clinical settings and has shown good interrater reliability (37, 38).

### 5.2 Automated MRI Volumetry

Automated volumetric analysis enables precise quantification of regional brain volumes, identifying characteristic PCA patterns such as predominant right parietal superior and occipital lobe atrophy (4, 42). Techniques like voxel-based morphometry (VBM) and cortical thickness analysis reveal significant gray matter reductions and assist in differential diagnosis. Combining automated methods and visual rating scales notably improved the explained variance in diagnostic predictions of cognitive impairment, especially regarding measures of posterior atrophy (43). Despite its advantages in objectivity and reproducibility, volumetric analysis remains less accessible due to higher costs and technical requirements compared to visual rating scales (Figure 2G).

## 6 PET imaging findings in PCA

Functional imaging with PET modalities reveals specific patterns of metabolism and pathology in PCA.

### 6.1 Fluorodeoxyglucose PET

FDG-PET typically shows hypometabolism in the parieto-occipital regions, mirroring structural degeneration and tau deposition, but not strongly correlated with amyloid pathology. This metabolic pattern corresponds to visuospatial deficits characteristic of PCA (44–46) (Figure 2H).

While primary visual cortex hypometabolism is more typical of Lewy body dementia (LBD) than it is of “typical” AD (47, 48), PCA also exhibits hypometabolism which is however, more asymmetric and extends into parietotemporo-occipital regions, with relative preservation of medial occipital metabolism. This creates the so-called ‘occipital tunnel sign’ on sagittal FDG-PET images, which occurs in both conditions (49).

Another distinguishing feature is the “cingulate island sign,” reflecting relative preservation of posterior cingulate metabolism, more commonly associated with Lewy body dementia (50).

### 6.2 Amyloid PET

Amyloid deposition appears diffusely across the neocortex, showing poor correlation with clinical symptoms or hypometabolic patterns, reinforcing the concept of amyloid as a disease marker rather than a clinical phenotype determinant (51, 52). Therefore, it does not distinguish PCA from amnestic AD.

### 6.3 Tau PET

Tau PET imaging shows selective retention predominantly in occipitoparietal areas — regions directly implicated in PCA clinical manifestations. Tau distribution correlates well with both hypometabolism and symptom severity, serving as a more accurate marker of clinical phenotype. High tau load consistently localizes to posterior brain regions in PCA (44, 51–53).

### 6.4 Advanced imaging techniques not incorporated into clinical practice

Diffusion tensor imaging (DTI) and functional magnetic resonance imaging (fMRI) methods have also been applied to PCA to investigate structural and functional connectivity, respectively. Although they are not routinely used in clinical practice, they have so far helped to better understand the neural correlates of PCA and suggest some distinctive features in regard to related conditions. For instance, as compared with amnestic AD patients, patients with PCA have different patterns of white matter connectivity change within the spectrum of AD, with the more significant differences in the right posterior regions in PCA patients (54). White matter connectivity may also distinguish PCA patients from LBD patients, in whom no significant white matter degeneration is seen (55). On the other hand, functional connectivity studies show considerable overlap between PCA and LBD patients, although some distinctive features may be seen, notably reduced within-network connectivity in the dorsal and ventral default-mode network in PCA patients (56).

## 7 Conclusion

PCA represents a critical but often underdiagnosed variant of AD. A structured and anatomically informed approach to neuroimaging interpretation is essential for early and accurate diagnosis. Knowledge of the visual system’s organization and careful evaluation of atrophy patterns, metabolic changes, and molecular imaging findings enable differentiation of PCA from other dementias and appropriate management planning.

As emerging disease-modifying treatments become more widely available, the early identification of atypical presentations like PCA will play an increasingly vital role in clinical practice.

## Footnotes

^1^Maia da Silva MN, James-Galton M, Green C, Plant GT. Homonymous Hemianopia in Posterior Cortical Atrophy: right-left Asymmetry, Progression over Time and Relationship to the Classical Neuropsychological Deficits. (In submission).

## References

[1] Cogan DG. Visual disturbances with focal progressive dementing disease. Am J Ophthalmol. (1985) 100:68–72. doi: 10.1016/s0002-9394(14)74985-2, PMID: 3893141

[2] Benson DF Davis RJ Snyder BD. Posterior cortical atrophy. Arch Neurol. (1988) 45:789–93. doi: 10.1001/archneur.1988.00520310107024, PMID: 3390033

[3] Jones D Pelak V Rogalski E. Atypical presentations of Alzheimer disease. Continuum. (2024) 30:1614–41. doi: 10.1212/CON.0000000000001504, PMID: 39620837 PMC12175143

[4] Lehmann M Crutch SJ Ridgway GR Ridha BH Barnes J Warrington EK. Cortical thickness and voxel-based morphometry in posterior cortical atrophy and typical Alzheimer’s disease. Neurobiol Aging. (2011) 32:1466–76. doi: 10.1016/j.neurobiolaging.2009.08.017, PMID: 19781814

[5] Alladi S Xuereb J Bak T Nestor P Knibb J Patterson K. Focal cortical presentations of Alzheimer’s disease. Brain. (2007) 130:2636–45. doi: 10.1093/brain/awm213, PMID: 17898010

[6] Chapleau M La Joie R Yong K Agosta F Allen IE Apostolova L. Demographic, clinical, biomarker, and neuropathological correlates of posterior cortical atrophy: an international cohort study and individual participant data meta-analysis. Lancet Neurol. (2024) 23:168–77. doi: 10.1016/S1474-4422(23)00414-3, PMID: 38267189 PMC11615965

[7] Renner JA Burns JM Hou CE McKeel DW Storandt M Morris JC. Progressive posterior cortical dysfunction: a clinicopathologic series. Neurology. (2004) 63:1175–80. doi: 10.1212/01.WNL.0000140290.80962.BF, PMID: 15477534

[8] Tang-Wai DF Graff-Radford NR Boeve BF Dickson DW Parisi JE Crook R. Clinical, genetic, and neuropathologic characteristics of posterior cortical atrophy. Neurology. (2004) 63:1168–74. doi: 10.1212/01.WNL.0000140289.18472.15, PMID: 15477533

[9] Levine DN Lee JM Fisher CM. The visual variant of Alzheimer’s disease: a clinicopathologic case study. Neurology. (1993) 43:305–13. doi: 10.1212/wnl.43.2.305, PMID: 8437694

[10] Abdi Z Yong KX Schott JM Gatt A Revesz T Crutch SJ. Pathological characterisation of posterior cortical atrophy in comparison with amnestic Alzheimer’s disease. Neuropathol Appl Neurobiol. (2025) 51:e70007. doi: 10.1111/nan.70007, PMID: 40174910 PMC11964714

[11] Dubois B Feldman HH Jacova C Hampel H Molinuevo JL Blennow K. Advancing research diagnostic criteria for Alzheimer’s disease: the IWG-2 criteria. Lancet Neurol. (2014) 13:614–29. doi: 10.1016/S1474-4422(14)70090-0, PMID: 24849862

[12] Koedam ELGE Lauffer V Van Der Vlies AE Van Der Flier WM Scheltens P Pijnenburg YAL. Early-versus late-onset Alzheimer’s disease: more than age alone. J Alzheimers Dis. (2010) 19:1401–8. doi: 10.3233/JAD-2010-1337, PMID: 20061618

[13] Snowden JS Stopford CL Julien CL Thompson JC Davidson Y Gibbons L. Cognitive phenotypes in Alzheimer’s disease and genetic risk. Cortex. (2007) 43:835–45. doi: 10.1016/S0010-9452(08)70683-X, PMID: 17941342

[14] Yong KXX Graff-Radford J Ahmed S Chapleau M Ossenkoppele R Putcha D. Diagnosis and Management of posterior cortical atrophy. Curr Treat Options Neurol. (2023) 25:23–43. doi: 10.1007/s11940-022-00745-0, PMID: 36820004 PMC9935654

[15] Crutch SJ Schott JM Rabinovici GD Murray M Snowden JS van der Flier WM. Consensus classification of posterior cortical atrophy on behalf of the Alzheimer’s association ISTAARTAtypical Alzheimer’s disease and associated syndromes professional interest area HHS public access. Alzheimers Dement. (2017) 13:870–84. doi: 10.1016/j.jalz.2017.01.014, PMID: 28259709 PMC5788455

[16] Maia da Silva MN Millington RS Bridge H James-Galton M Plant GT. Visual dysfunction in posterior cortical atrophy. Front Neurol. (2017) 8:389. doi: 10.3389/fneur.2017.00389, PMID: 28861031 PMC5561011

[17] Geldmacher DS. Treatment of Alzheimer disease. Continuum. (2024) 30:1823–44. doi: 10.1212/CON.0000000000001503, PMID: 39620846

[18] Pelak VS. Disorders of higher-order visual function. Continuum. (2025) 31:543–65. doi: 10.1212/CON.0000000000001555, PMID: 40179408

[19] Prasad S Dinkin M. *Higher cortical visual disorders*. (2019). Available online at: http://journals.lww.com/continuum. 10.1212/CON.0000000000000774 31584540

[20] Prasad S Galetta SL. Anatomy and physiology of the afferent visual system. Handb Clin Neurol. (2011) 102. doi: 10.1016/B978-0-444-52903-9.00007-8, PMID: 21601061

[21] Felleman DJ Van Essen DC. Distributed hierarchical processing in the primate cerebral cortex In: Felleman DJ, editor. Cerebral cortex, vol. 1. New York: Oxford University Press (1991) 10.1093/cercor/1.1.1-a 1822724

[22] Maia da Silva MN James-Galton M Pinho JD Sethi VV Plant GT. Homonymous hemianopia in posterior cortical atrophy: an enigma. J Neurol Sci. (2015) 357:e132. doi: 10.1016/j.jns.2015.08.425 PMC13008642 41884339

[23] Crutch SJ Lehmann M Schott JM Rabinovici GD Rossor MN Fox NC. Posterior cortical atrophy. Lancet Neurol. (2012) 11:170–8. doi: 10.1016/S1474-4422(11)70289-7, PMID: 22265212 PMC3740271

[24] Rizzo M Vecera SP. Psychoanatomical substrates of Bálint’s syndrome. J Neurol Neurosurg Psychiatry. (2002) 72:162–78. doi: 10.1136/jnnp.72.2.162, PMID: 11796765 PMC1737727

[25] Barton JJS Cherkasova M O’Connor M. Covert recognition in acquired and developmental prosopagnosia. Neurology. (2001) 57:1161–8. doi: 10.1212/WNL.57.7.1161, PMID: 11591830

[26] Akeret K van Niftrik CHB Sebök M Muscas G Visser T Staartjes VE. Topographic volume-standardization atlas of the human brain. Brain Struct Funct. (2021) 226:1699–711. doi: 10.1007/s00429-021-02280-1, PMID: 33961092 PMC8203509

[27] Kennedy DN Lange N Makris N Bates J Meyer J Caviness VS. Gyri of the human neocortex: an MRI-based analysis of volume and variance. Cereb Cortex. (1998) 8:372–84. doi: 10.1093/cercor/8.4.372, PMID: 9651132

[28] Naidich TP Castillo M Cha S Smirniotopoulos JG. Imaging of the brain. Imaging Brain. (2012) 10:41. doi: 10.3928/0098-9134-19840901-14

[29] Flores LP. Occipital lobe morphological anatomy: anatomical and surgical aspects. Arq Neuropsiquiatr. (2002) 60:566–71. doi: 10.1590/s0004-282x2002000400010, PMID: 12244393

[30] Alves RV Ribas GEC Párraga RG De Oliveira EO. The occipital lobe convexity sulci and gyri: laboratory investigation. J Neurosurg. (2012) 116:1014–23. doi: 10.3171/2012.1.JNS11978, PMID: 22339163

[31] Koutsarnakis C Komaitis S Drosos E Kalyvas AV Skandalakis GP Liakos F. Mapping the superficial morphology of the occipital lobe: proposal of a universal nomenclature for clinical and anatomical use. Neurosurg Rev. (2021) 44:335–50. doi: 10.1007/s10143-019-01212-2, PMID: 31758336

[32] Hinds O Polimeni JR Rajendran N Balasubramanian M Amunts K Zilles K. Locating the functional and anatomical boundaries of human primary visual cortex. NeuroImage. (2009) 46:915–22. doi: 10.1016/j.neuroimage.2009.03.036, PMID: 19328238 PMC2712139

[33] Funkhouser EB. The visual cortex, its localization, histological structure, and physiological function. J Exp Med. (1915) 21:617–28. doi: 10.1084/jem.21.6.617, PMID: 19867896 PMC2125292

[34] Barbier EL Marrett S Danek A Vortmeyer A Van Gelderen P Duyn J. Imaging cortical anatomy by high-resolution MR at 3.0T: detection of the stripe of Gennari in visual area 17. Magn Reson Med. (2002) 48:735–8. doi: 10.1002/mrm.10255, PMID: 12353293

[35] Shir D Graff-Radford J Machulda MM Pham NTT Jack CR Lowe VJ. Posterior cortical atrophy: primary occipital variant. Eur J Neurol. (2022) 29:2138–43. doi: 10.1111/ene.15327, PMID: 35298068 PMC9703695

[36] Millington RS James-Galton M Maia Da Silva MN Plant GT Bridge H. Lateralized occipital degeneration in posterior cortical atrophy predicts visual field deficits. Neuroimage Clin. (2017) 14:242–9. doi: 10.1016/j.nicl.2017.01.012, PMID: 28180083 PMC5288489

[37] Harper L Barkhof F Fox NC Schott JM. Using visual rating to diagnose dementia: a critical evaluation of MRI atrophy scales. In. J Neurol Neurosurg Psychiatry. (2015) 86:1225–33. doi: 10.1136/jnnp-2014-310090, PMID: 25872513

[38] Pasquier F Leys D Weerts JGE Mounier-Vehier F Barkhof F Scheltens P. Inter-and intraobserver reproducibility of cerebral atrophy assessment on mri scans with hemispheric infarcts. Eur Neurol. (1996) 36:268–72. doi: 10.1159/000117270, PMID: 8864706

[39] Dziedzic TA Bala A Balasa A Olejnik A Marchel A. Anatomy of the occipital lobe using lateral and posterior approaches: a neuroanatomical study with a neurosurgical perspective on intraoperative brain mapping. Folia Morphol (Poland). (2023) 82:7–16. doi: 10.5603/FM.a2021.0140, PMID: 35037696

[40] Koedam ELGE Lehmann M Van Der Flier WM Scheltens P Pijnenburg YAL Fox N. Visual assessment of posterior atrophy development of a MRI rating scale. Eur Radiol. (2011) 21:2618–25. doi: 10.1007/s00330-011-2205-4, PMID: 21805370 PMC3217148

[41] Fumagalli GG Basilico P Arighi A Mercurio M Scarioni M Carandini T. Parieto-occipital sulcus widening differentiates posterior cortical atrophy from typical Alzheimer disease. Neuroimage Clin. (2020) 28:102453. doi: 10.1016/j.nicl.2020.102453, PMID: 33045537 PMC7559336

[42] Möller C Van Der Flier WM Versteeg A Benedictus MR Wattjes MP Koedam ELGM. Quantitative regional validation of the visual rating scale for posterior cortical atrophy. Eur Radiol. (2014) 24:397–404. doi: 10.1007/s00330-013-3025-5, PMID: 24092044

[43] Persson K Barca ML Edwin TH Cavallin-Eklund L Tangen GG Rhodius-Meester HFM. Regional MRI volumetry using NeuroQuant versus visual rating scales in patients with cognitive impairment and dementia. Brain Behav. (2024) 14:e3397. doi: 10.1002/brb3.3397, PMID: 38600026 PMC10839122

[44] Ossenkoppele R Schonhaut DR Baker SL O’Neil JP Janabi M Ghosh PM. Tau, amyloid, and hypometabolism in a patient with posterior cortical atrophy. Ann Neurol. (2015) 77:24321. doi: 10.1002/ana.24321 PMC4382124 25448043

[45] Singh TD Josephs KA Machulda MM Drubach DA Apostolova LG Lowe VJ. Clinical, FDG and amyloid PET imaging in posterior cortical atrophy. J Neurol. (2015) 262:1483–92. doi: 10.1007/s00415-015-7732-5, PMID: 25862483 PMC4469094

[46] Strom A Iaccarino L Edwards L Lesman-Segev OH Soleimani-Meigooni DN Pham J. Cortical hypometabolism reflects local atrophy and tau pathology in symptomatic Alzheimer’s disease. Brain. (2022) 145:713–28. doi: 10.1093/brain/awab294, PMID: 34373896 PMC9014741

[47] Fujishiro H Iseki E Kasanuki K Murayama N Ota K Suzuki M. Glucose hypometabolism in primary visual cortex is commonly associated with clinical features of dementia with Lewy bodies regardless of cognitive conditions. Int J Geriatr Psychiatry. (2012) 27:1138–46. doi: 10.1002/gps.2836, PMID: 22250011

[48] Minoshima S Foster NL Sima AAF Frey KA Albin RL Kuhl DE. Alzheimer’s disease versus dementia with Lewy bodies: cerebral metabolic distinction with autopsy confirmation. Ann Neurol. (2001) 50:358–65. doi: 10.1002/ana.1133, PMID: 11558792

[49] Sawyer DM Kuo PH. “Occipital tunnel” sign on FDG PET for differentiating dementias. Clin Nucl Med. (2018) 43:e59–61. doi: 10.1097/RLU.0000000000001925, PMID: 29232244

[50] Feng LR Vogel A Mellergaard C Waldemar G Hasselbalch SG Law I. Clinical validation of the cingulate island sign visual rating scale in dementia with Lewy bodies. J Neurol Sci. (2023) 451:120719. doi: 10.1016/j.jns.2023.120719, PMID: 37421880

[51] Lehmann M Ghosh PM Madison C Laforce R Corbetta-Rastelli C Weiner MW. Diverging patterns of amyloid deposition and hypometabolism in clinical variants of probable Alzheimer’s disease. Brain. (2013) 136:844–58. doi: 10.1093/brain/aws327, PMID: 23358601 PMC3580269

[52] Ossenkoppele R Schonhaut DR Schöll M Lockhart SN Ayakta N Baker SL. Tau PET patterns mirror clinical and neuroanatomical variability in Alzheimer’s disease. Brain. (2016) 139:1551–67. doi: 10.1093/brain/aww027, PMID: 26962052 PMC5006248

[53] La Joie R Visani AV Lesman-Segev OH Baker SL Edwards L Iaccarino L. Association of APOE4 and clinical variability in Alzheimer disease with the pattern of tau- and amyloid-PET. Neurology. (2021) 96:e650–61. doi: 10.1212/WNL.0000000000011270, PMID: 33262228 PMC7884991

[54] Torso M Ahmed S Butler C Zamboni G Jenkinson M Chance S. Cortical diffusivity investigation in posterior cortical atrophy and typical Alzheimer's disease. J Neurol. (2021) 268:227–39. doi: 10.1007/s00415-020-10109-w, PMID: 32770413 PMC7815619

[55] Singh NA Graff-Radford J Machulda MM Pham NTT Schwarz CG Reid RI. Diffusivity changes in posterior cortical atrophy and Logopenic progressive aphasia: a longitudinal diffusion tensor imaging study. J Alzheimers Dis. (2023) 94:709–25. doi: 10.3233/JAD-221217, PMID: 37302032 PMC10785680

[56] Singh NA Goodrich AW Graff-Radford J Machulda MM Sintini I Carlos AF. Altered structural and functional connectivity in posterior cortical atrophy and dementia with Lewy bodies. NeuroImage. (2024) 290:120564. doi: 10.1016/j.neuroimage.2024.120564, PMID: 38442778 PMC11019668

